# Regional Lung Recruitability During Pneumoperitoneum Depends on Chest Wall Elastance – A Mechanical and Computed Tomography Analysis in Rats

**DOI:** 10.3389/fphys.2018.00920

**Published:** 2018-07-13

**Authors:** Lucia Comuzzi, Mariana B. de Abreu, Gabriel C. Motta-Ribeiro, Renata T. Okuro, Thiago Barboza, Niedja Carvalho, Umberto Lucangelo, Alysson R. Carvalho, Walter A. Zin

**Affiliations:** ^1^Laboratory of Respiration Physiology, Carlos Chagas Filho Institute of Biophysics, Universidade Federal do Rio de Janeiro, Rio de Janeiro, Brazil; ^2^Department of Perioperative Medicine, Intensive Care and Emergency, Università degli Studi di Trieste, Trieste, Italy; ^3^Laboratory of Pulmonary Engineering, Alberto Luiz Coimbra Institute of Post-Graduation and Engineering Research, Universidade Federal do Rio de Janeiro, Rio de Janeiro, Brazil; ^4^National Center for Structural Biology and Bioimaging, Universidade Federal do Rio de Janeiro, Rio de Janeiro, Brazil

**Keywords:** lung recruitability, PEEP, chest wall elastance, pneumoperitoneum, computed tomography scanning

## Abstract

**Background:** Laparoscopic surgery with pneumoperitoneum increases respiratory system elastance due to the augmented intra-abdominal pressure. We aim to evaluate to which extent positive end-expiratory pressure (PEEP) is able to counteract abdominal hypertension preventing progressive lung collapse and how rib cage elastance influences PEEP effect.

**Methods:** Forty-four Wistar rats were mechanically ventilated and randomly assigned into three groups: control (CTRL), pneumoperitoneum (PPT) and pneumoperitoneum with restricted rib cage (PPT-RC). A pressure-volume (PV) curve followed by a recruitment maneuver and a decremental PEEP trial were performed in all groups. Thereafter, animals were ventilated using PEEP of 3 and 8 cmH_2_O divided into two subgroups used to evaluate respiratory mechanics or computed tomography (CT) images. In 26 rats, we compared respiratory system elastance (E_rs_) at the two PEEP levels. In 18 animals, CT images were acquired to calculate total lung volume (TLV), total volume and air volume in six anatomically delimited regions of interest (three along the cephalo-caudal and three along the ventro-dorsal axes).

**Results:** PEEP of minimal E_rs_ was similar in CTRL and PPT groups (3.8 ± 0.45 and 3.5 ± 3.89 cmH_2_O, respectively) and differed from PPT-RC group (9.8 ± 0.63 cmH_2_O). Chest restriction determined a right- and downward shift of the PV curve, increased E_rs_ and diminished TLV and lung aeration. Increasing PEEP augmented TLV in CTRL group (11.8 ± 1.3 to 13.6 ± 2 ml, *p* < 0.05), and relative air content in the apex of PPT group (3.5 ± 1.4 to 4.6 ± 1.4% TLV, *p* < 0.03) and in the middle zones in PPT-RC group (21.4 ± 1.9 to 25.3 ± 2.1% TLV cephalo-caudally and 18.1 ± 4.3 to 22.0 ± 3.3% TLV ventro-dorsally, *p* < 0.005).

**Conclusion:** Regional lung recruitment potential during pneumoperitoneum depends on rib cage elastance, reinforcing the concept of PEEP individualization according to the patient’s condition.

## Introduction

Laparoscopic surgery has been increasingly used as an alternative to open surgery for its well-known post-operative benefits. However, it is associated to intra-operative respiratory impairment (Valenza et al., 2010). Mechanical ventilation management is a challenge for the anesthetist in this scenario of intra-abdominal hypertension, because it is not clear how the modification of the ventilation parameters affects the different components of the respiratory system.

The respiratory system is composed of two elastic elements in series: the lung and the chest wall. The latter, in turn, is made up of two parallel components: the rib cage and the diaphragm, which is also part of the abdominal wall. Disregarding airflow resistances, the positive pressure applied to the respiratory system during mechanical ventilation distends all these elements. How the applied pressure is distributed within the respiratory system depends on the compliance of each single element (Cortes-Puentes et al., 2015) and its distribution within the lungs depends on the compliance of the lung zones, i.e., regional compliance (Mutoh et al., 1991; Lowhagen et al., 2010).

The production of a pneumoperitoneum with an intra-abdominal pressure (P_ab_) above the physiological is required to perform laparoscopic surgery. Andersson et al. (2005) showed by CT scans that pneumoperitoneum causes a cranial displacement of the diaphragm and stiffens it. Moreover, the dependent lung zones are more atelectatic and there is a reduction in pulmonary volumes (Andersson et al., 2005). As a result, respiratory system elastance (E_rs_) increases due to higher chest wall (E_w_) and lung (E_L_) elastances (Mutoh et al., 1991; Fahy et al., 1995; Moreira et al., 1997; Valenza et al., 2007a,b; Maracajá-Neto et al., 2009; Futier et al., 2010; Formenti et al., 2012; Regli et al., 2012; Runck et al., 2012; Cinnella et al., 2013; Cortes-Puentes et al., 2013, 2015; Loring et al., 2014). PEEP triggers different outcomes in conditions of heterogeneous regional E_L_ and E_w_, e.g., intra-abdominal hypertension (Formenti et al., 2012; Regli et al., 2012; Runck et al., 2012; Cortes-Puentes et al., 2015). However, to our knowledge, the regional PEEP distribution within the lungs during pneumoperitoneum has not been so far described.

We aimed to evaluate the recruitment potential of two different PEEP levels in rats with modified chest wall compliance by pneumoperitoneum alone and pneumoperitoneum plus restricted chest. The assessment was done globally, by means of respiratory mechanics, and regionally, by quantitative CT imaging. We hypothesized that the more restricted is the chest, the larger is the PEEP recruitment potential.

## Materials and Methods

### Animals and Study Groups

Forty-eight male Wistar rats (330–430 g) were used. All received human care in compliance with the National Institutes of Health Guide for the Care and Use of Laboratory Animals (NIH Publications No. 8023, revised 1978), and National Council for Controlling Animal Experimentation, Ministry of Science, Technology and Innovation (CONCEA/MCTI), Brazil. The Ethics Committee on the Use of Animals, Health Sciences Centre, Federal University of Rio de Janeiro approved the experimental protocol (103/2013).

Rats were housed in groups of five animals per cage (Makrolon polycarbonate Type IV) and food and water were provided *ad libitum.* They were randomly divided using sealed envelopes into three groups: control (CTRL), pneumoperitoneum (PPT) and pneumoperitoneum with restricted chest (PPT-RC). Twelve animals were allocated in CTRL group and 16 in each of the other two groups. Each group was further divided into two subgroups used to evaluate respiratory mechanics or CT images. Six animals from each group underwent whole lung CT scans in a preclinical micro-CT/PET/SPECT scanner (Tri-Modality FLEX Triumph Pre-Clinical Imaging System, Gamma Medica-Ideas, Northridge, CA, United States). The remaining 26 animals were used for measurement of global respiratory system mechanics (6 in CTRL and 10 in PPT and PPT-RC groups).

Sample size was determined to reach a power of 80% and a significance level of 1.67% (5% corrected for the three-group comparison), with an effect size of 0.9. Only 12 rats were allocated to CTRL group because it was expected to be more homogenous.

### Experimental Protocol

Animals were anesthetized with isoflurane 2.5% vol with a calibrated vaporizer. A 24-G catheter was indwelled into a tail vein and a 20-G catheter into the right carotid artery. Then, the rats were tracheotomized, paralyzed with pancuronium bromide (0.2 mg/kg IV) and mechanically ventilated (Inspira ASV, Harvard Apparatus, Holliston, MA, United States) in volume-controlled ventilation mode with tidal volume (V_T_) of 8 ml/kg, respiratory rate (RR) of 70 breaths/min, inspiratory to expiratory time ratio (I:E) of 1:2, PEEP of 5 cmH_2_O and inspired oxygen fraction ratio (FiO_2_) of 0.5.

Afterward, 1 ml of a colloid solution (6% hydroxyethyl starch 130/0.42, Centralvet, Vinhedo, SP, Brazil) was administered intravenously and a recruitment maneuver was performed by setting PEEP at 15 cmH_2_O for five consecutive breathing cycles. After the procedure, PEEP was set at 5 cmH_2_O, and, after 5 min, arterial blood was collected for gas analysis (T0, i-STAT with EG4^+^ Cartridge, Abbott, Chicago, IL, United States). If arterial oxygen partial pressure to inspired oxygen fraction (PaO_2_/FiO_2_) was ≥400, the experimental protocol started. If not, the maneuver was repeated with a PEEP of 20 cmH_2_O and if PaO_2_/FiO_2_ was ≥400 the experimental protocol began. If after the recruitment maneuvers PaO_2_/FiO_2_ remained <400, the animal was cast aside.

Before pneumoperitoneum production, 2 ml of colloid were administered intravenously in the PPT and PPT-RC groups. Then, a 16-G catheter was inserted through and fixed to the abdominal wall, and nitrogen was used for abdominal insufflation. We used an inert gas because during CT scan it was not possible to maintain the animal connected to the insufflation system and adjust gas pressure. The target P_ab_ was 16–20 cmH_2_O. In PPT–RC group, chest restriction was performed with a sphygmomanometer (number 4) wrapped around the thorax of the animal and inflated until a pressure of 12–15 cmH_2_O was reached. Two pressure transducers (UT-PDP-50, SCIREQ, Montreal, QC, Canada), connected to a computer, controlled P_ab_ and the insufflation pressure of the sphygmomanometer.

Thereafter, the inspiratory limb of pressure-volume (PV) curves was obtained with a low-flow inflation by adjusting PEEP to 0 cmH_2_O, RR to 5 breaths/min, I:E ratio to 4:1 and V_T_ to 30 mL/kg. The resulting inspiratory flow was <200 ml/min. Four PV curves were performed and that presenting a stable peak airway pressure (*circa* 35 cmH_2_O) was considered for further analysis (Carvalho et al., 2013).

Right after gathering the PV curve, a recruitment maneuver with the formerly successful PEEP (15 or 20 cmH_2_O) and a decremental PEEP trial were performed. For the latter, PEEP was set at 10 cmH_2_O and decreased to 1 cmH_2_O in unitary steps lasting 30 s each (Carvalho et al., 2013).

All animals were then ventilated using two different PEEPs, 3 and 8 cmH_2_O. Firstly, ventilation was performed with PEEP of either 3 or 8 cmH_2_O for 10 min, and, then, they were ventilated for another 10 min with the other PEEP. The sequence was determined in a random fashion using sealed envelopes. Before applying each PEEP, all animals were maintained at PEEP = 1 cmH_2_O for 5 min, to maintain volume history. At the end of the experiment, euthanasia was performed by sectioning the inferior vena cava and abdominal aorta under deep anesthesia (isoflurane 5% vol).

### Data Acquisition and Processing

Airway pressure (P_aw_) and flow (V′) were continuously measured using a heated pneumotachograph (8430B, Hans Rudolph, Shawnee, KS, United States) connected between the endotracheal tube and the Y-piece of the ventilatory circuit. The pneumotachograph was connected to two differential pressures transducers, one for P_aw_ (UT-PDP-50, SCIREQ, Montreal, QC, Canada) and another for V′ (UT-PDP-02, SCIREQ, Montreal, QC, Canada) measurements. P_aw_ and V′ signals were low-pass filtered at 30 Hz, digitized at 1000 Hz using a 14-bit analog-to-digital converter (NI-6009, National Instruments, Austin, TX, United States) and recorded with a built-purpose routine written in LabVIEW (National Instruments, Austin, TX, United States). All transducers were calibrated before the experiments.

V_T_ was calculated by numerical integration of V′ and P_aw_ was fitted on a breath-by-breath basis to a linear single-compartment model (Eq. 1) for the estimation of respiratory system elastance (E_rs_):

(1) $$Paw(t)=Ers\cdot V(t)+Rrs\cdot V^{\prime }(t)+EEP$$

where R_rs_ is the respiratory system resistance, EEP is the end-expiratory pressure at zero V′ and volume (V) and t is time.

The mechanical parameters were estimated considering the mean value from the last 20 cycles at the end of each PEEP step during PEEP titration and 40 cycles at each PEEP used during the ventilation protocol (PEEP 1, 3, and 8 cmH_2_O). The PEEP at minimal E_rs_ (PEEPmin-_Ers_) was calculated as the minimum of a third degree polynomial fitted to E_rs_ versus PEEP curve during the titration maneuver. Additionally, we compared E_rs_ using PEEP = 3 vs. 8 cmH_2_O in each group and among groups.

### Computed Tomography Scan Acquisition and Processing

Computed tomography scan was performed with a small animal micro-PET/SPECT/CT scan (Tri-Modality FLEX Triumph Pre-Clinical Imaging System, Gamma Medica-Ideas, Northridge, CA, United States). The acquisition protocol was based on 5-mm-thick axial slices, 1.5x collimation, FOV 78.92, Binning 2x2, 3 frames of 1024 slices, 75 kVp and 135 μAs. The procedure for each CT scan lasted about 10 min.

In all images, lung parenchyma was segmented semi-automatically using a threshold and growing region algorithm with multiple seeds in Osirix software (Pixmeo, Geneva, Switzerland). Data were then exported as DICOM files for images, and HDR+IMG for regions of interest (ROI), to be later processed in MATLAB (MathWorks, Natick, MA, United States) using a custom code.

We divided the lungs in three ROI along the cranio-caudal axis (apex, middle, and base) and three ROI along the ventro-dorsal axis (ventral, middle, and dorsal). The division obeyed visually identified anatomical landmarks: the carina and the heart apex for the cranio-caudal axis, and the top of the heart (considering the heart zone closest to the sternum in a lateral view) and the top of the hilum (approximately the entrance of the airways into the lungs) for the ventro-dorsal axis. We calculated and compared TLV using PEEP = 3 vs. 8 cmH_2_O in each group and also among the groups. Additionally, we calculated and compared the total volume and the air volume of each ROI using PEEP = 3 vs. 8 cmH_2_O in each group. To account for differences in lung dimensions, the volumes of the ROIs were normalized by TLV.

Finally, we processed CT images to describe the lung density distribution, expressed in Hounsfield units, in the three groups using PEEP = 3 and 8 cmH_2_O. The integral of the curves corresponds to TLV.

### Statistical Analysis

Intra-group comparisons were performed with paired *t*-test and inter-group comparisons with one-way ANOVA. Multiple comparisons were corrected with the Bonferroni–Holm’s method. All analysis were performed in MATLAB (MathWorks, Natick, MA, United States) considering α = 5%.

## Results

Three animals of PPT-RC group were excluded because baseline PaO_2_/FiO_2_ was ≤400 mmHg after the second lung recruitment maneuver and one rat of PPT group died during micro-CT scanning. These rats were replaced by animals from the same batch. We were unable to properly reconstruct the CT image in one CTRL animal, therefore only images of five rats were analyzed in that group.

### Respiratory Mechanics

**FIGURE 1:**
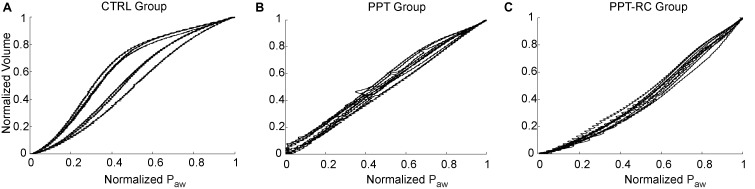
Inspiratory limb of pressure versus volume (PV) curves in the three experimental groups **(A–C)** obtained with the low flow inflation. Each line represents one animal. Airway pressure (P_aw_) and volume are normalized considering the maximum and minimum value of each animal. CTRL, control group; PPT, animals with pneumoperitoneum; PPT-RC, animals with pneumoperitoneum and rib cage restriction.

Normalized inspiratory PV curves of all animals are shown in **Figure 1**. CTRL group (**Figure 1A**) showed sigmoidal PV curves with discreet lower and evident upper inflection points, suggesting the predominance of overdistension in relation to recruitment. In the PPT group, linear PV curves were observed (**Figure 1B**), whereas in the PPT-RC group the upward volume/pressure concavity suggests a progressive recruitment throughout inflation (**Figure 1C**). TLCs were 10.9 ± 1.0, 9.1 ± 1.5, and 7.3 ± 2.2 ml in CTRL, PPT, and PPT-RC groups, respectively.

**FIGURE 2:**
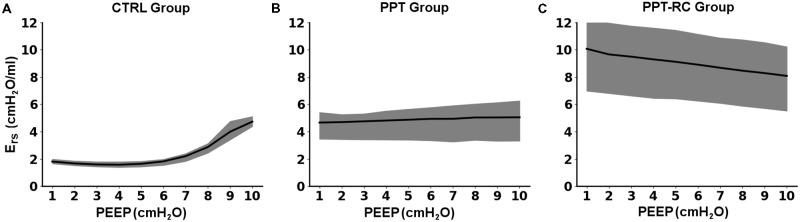
Changes in respiratory system elastance (E_rs_) as a function of PEEP during the decremental PEEP trial in the three experimental groups **(A–C)**. Black line and gray band represent mean values and standard deviations, respectively. CTRL, control group; PPT, animals with pneumoperitoneum; PPT-RC, animals with pneumoperitoneum and rib cage restriction.

**Figure 2** presents E_rs_ versus PEEP in each group during the decremental PEEP trial. In CTRL group (**Figure 2A**) E_rs_ progressively fell with decreasing PEEP up to a PEEP of 3.8 ± 0.4 cmH_2_O. Then, a slight increase in E_rs_ was observed, which resulted in a J-shaped profile. In PPT group (**Figure 2B**) E_rs_ was almost constant throughout the entire PEEP-trial (minimal E_rs_ at PEEP of 3.5 ± 3.89 cmH_2_O), whereas in the PPT-RC group (**Figure 2C**) E_rs_ increased while PEEP decreased reaching a minimal close to the highest tested PEEP at 9.8 ± 0.6 cmH_2_O.

**FIGURE 3:**
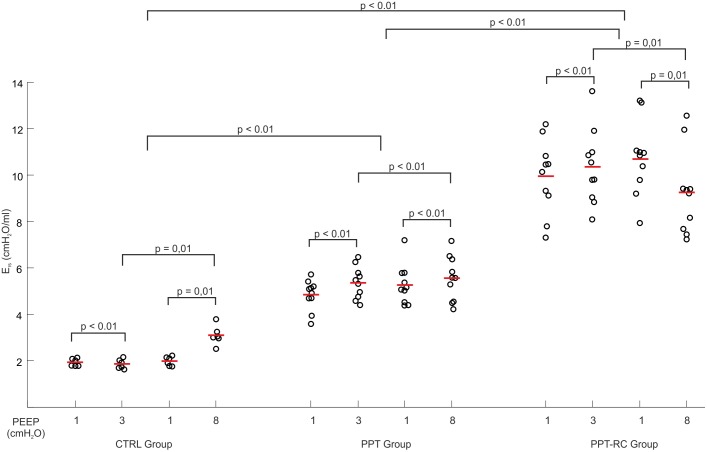
Changes in respiratory system elastance (E_rs_) as a function of PEEP during the ventilation protocol in the three groups. Each open circle depicts E_rs_ of a single animal, the red lines represent mean E_rs_. *p*-Values of different comparisons are shown. CTRL, control group; PPT, animals with pneumoperitoneum; PPT-RC, animals with pneumoperitoneum and rib cage restriction.

During the ventilation protocol, E_rs_ increased progressively with increasing chest restriction, independently of the PEEP applied (**Figure 3**). E_rs_ was higher in PEEP = 8 cmH_2_O than in PEEP = 3 cmH_2_O in groups CTRL and PPT, and smaller in PPT-RC group. E_rs_ was smaller in PEEP = 3 cmH_2_O than in PEEP = 1 cmH_2_O in CTRL group (without clinical relevance) and higher in the other two groups. Elastance was higher in PEEP = 8 cmH_2_O than in PEEP = 1 cmH_2_O in CTRL and PPT groups but diminished in PPT-RC group (**Figure 3**). In all groups the two measurements under PEEP = 1 cmH_2_O did not differ.

### CT Scan Images

**FIGURE 4:**
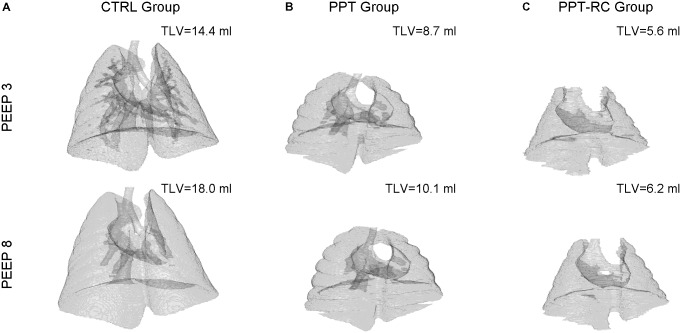
Examples of 3D lung surface reconstruction from CT scan images. For each group (columns **A–C**) a reconstruction at PEEPs = 3 (upper panels) and 8 cmH_2_O (lower panels), and the corresponding TLVs, are shown. Magnification is the same in all instances. CTRL, control group; PPT, animals with pneumoperitoneum; PPT-RC, animals with pneumoperitoneum and rib cage restriction.

**Figure 4** depicts examples of lung surface reconstructions obtained by processing CT scan images and the corresponding TLV for each group and PEEP.

**FIGURE 5:**
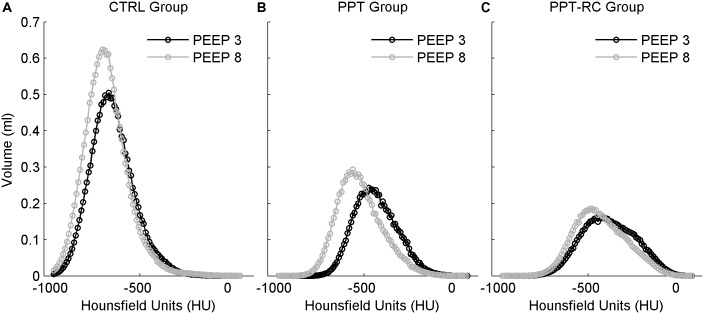
Histograms of mean lung density distribution, expressed in Hounsfield units, in the three groups **(A–C)**. Smaller values indicate lower density, thus more air content. Black circles: PEEP = 3 cmH_2_O, gray circles: PEEP = 8 cmH_2_O. The integral of the curves corresponds to TLV. CTRL, control group; PPT, animals with pneumoperitoneum; PPT-RC, animals with pneumoperitoneum and rib cage restriction.

**Figure 5** represents histograms showing mean lung density, which is correlated with lung aeration (Gattinoni et al., 1987), expressed in Hounsfield units, in the three groups at PEEPs = 3 and 8 cmH_2_O. Aeration has a unimodal distribution in all cases. With increasing chest restriction, a rightward shift of the curves, i.e., a decreasing aeration can be seen.

Total lung volume decreased progressively with increasing chest wall restriction, independently of the PEEP used (*p* < 0.001 in all comparisons). Increasing PEEP augmented TLV in CTRL group (11.8 ± 1.3 to 13.6 ± 2 ml, *p* = 0.036), while in the other groups TLV did not change (PPT group: 7.3 ± 0.7 and 8.1 ± 1.4 ml, PPT-RC group: 6.1 ± 0.7 and 6.2 ± 0.8 ml, *p* = 0.09 and 0.66, respectively). On the average, the percentage variations of TLV from PEEP 3 to 8 cmH_2_O were +15.2, +11, and +1.6% in CTRL, PPT and PPT-RC groups, respectively.

**FIGURE 6:**
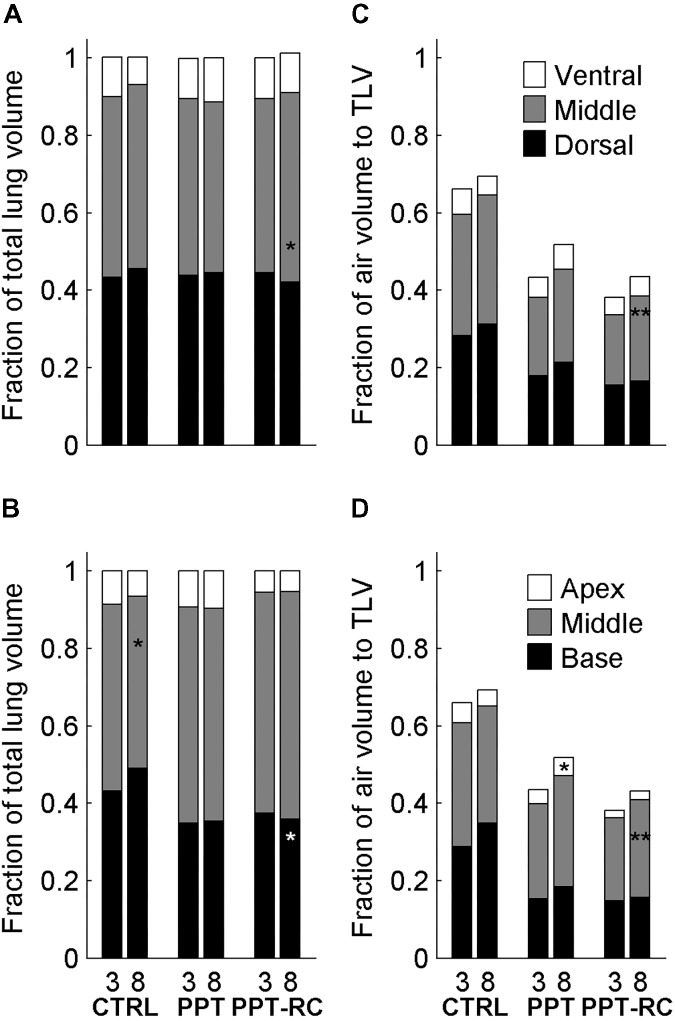
Fractional contribution of the volume of the regions of interest (ROI, three along the ventro-dorsal and three along the cephalocaudal axes) to TLV in the three groups at PEEP = 3 and 8 cmH_2_O **(A,B)** and fractional contribution of the ROI’s air volume to TLV in the three groups at PEEP = 3 and 8 cmH_2_O (**C,D**). Asterisks indicate statistically significant intra-group differences between PEEP = 3 and 8 cmH_2_O in each zone (^∗^*p* < 0.05, ^∗∗^*p* < 0.01). CTRL, control group; PPT, animals with pneumoperitoneum; PPT-RC, animals with pneumoperitoneum and rib cage restriction.

In addition, we analyzed the volume and the density of six ROIs, considering the cephalo-caudal (apex, middle, and base) and the ventro-dorsal (ventral, middle, and dorsal) axes. **Figure 6** shows the fractional contribution of total volume of each ROI to TLV (**Figures 6A,B**) and the fractional contribution of the air content of each ROI to TLV (**Figures 6C,D**), at both PEEP levels in each group. With increasing PEEP, the middle cephalo-caudal zone presented a smaller contribution to TLV in CTRL group (48.2 ± 5 to 44.4 ± 4.8%, *p* = 0.013); in PPT group, the apex air content exhibited a higher contribution to TLV with PEEP = 8 cmH_2_O (3.5 ± 1.4 to 4.6 ± 1.4%, *p* = 0.027); in PPT-RC group, the base presented a reduced contribution (37.5 ± 6.1 to 35.9 ± 5.9%, *p* = 0.023) and the middle ventro-dorsal zone an increased contribution to TLV with PEEP = 8 cmH_2_O (45 ± 7.2 to 48.9 ± 6.8%, *p* = 0.023). Moreover, in PPT-RC group the middle ventro-dorsal (18.1 ± 4.3 to 22 ± 3.3%, *p* = 0.003) and cephalo-caudal zones (21.4 ± 1.9 to 25.3 ± 2.1%, *p* = 0.003) increased their air volume relative to TLV under PEEP = 8 cmH_2_O.

## Discussion

The effects of pneumoperitoneum and PEEP on respiratory mechanics and aeration are well-known (Fahy et al., 1995; Moreira et al., 1997; Andersson et al., 2005; Valenza et al., 2007a,b; Maracajá-Neto et al., 2009; Futier et al., 2010; Formenti et al., 2012; Regli et al., 2012; Runck et al., 2012; Cinnella et al., 2013; Cortes-Puentes et al., 2013; Loring et al., 2014; Cortes-Puentes et al., 2015). However, there is no previous investigation about the consequences of different PEEP levels in the presence of pneumoperitoneum and variable E_w_ on respiratory mechanics and lung regional aeration.

The restriction of the ribcage was included in our study for three reasons: firstly, rats have naturally a more compliant chest wall than humans (Loring et al., 2010); secondly, we wanted to homogenize ribcage elastance among rats; thirdly, we believed that further increasing E_w_ would improve the effect of PEEP on lung recruitability.

Our main findings were: (1) the more restricted is the chest wall, the higher is E_rs_ and the lower is TLV, independently of PEEP; (2) PEEP of minimal E_rs_ is higher in the face of ribcage restriction; (3) accordingly, lung recruitability during intra-abdominal hypertension induced by pneumoperitoneum depends on the elastance of the ribcage.

### PV Curves

The PV curves of CTRL group showed the classical shape of rats’ PV curves, with a lower and a more distinct upper corner points (Martin-Lefèvre et al., 2001; Loring et al., 2010).

Restricting the entire chest wall, i.e., rib cage and abdominal compartments (PPT-RC group), led to a decrease in the slope of the curve, the disappearance of the upper corner, and a decreased final volume, in accordance with previous findings in rats with restricted ribcage (Loring et al., 2010).

Pneumoperitoneum alone (PPT group) produced similar and less important curve alterations, as previously reported (Mutoh et al., 1991; Valenza et al., 2007a,b; Runck et al., 2012; Loring et al., 2014). Moreover, the lower corner disappeared. This may be due to the high compliance of the rib cage that is expanded by PEEP, thus consuming pressure that would be otherwise available for lung recruitment. This supports our hypothesis about the role of the rib cage in determining the recruitment potential of PEEP in the presence of pneumoperitoneum.

### PEEP Titration Curves and Ventilation Protocol: PEEP = 3 Versus 8 cmH_2_O

We compared two PEEP levels. The lower level (PEEP = 3 cmH_2_O) was chosen because it is close to the mean value of PEEPmin-_Ers_ in rats (Carvalho et al., 2013; Camilo et al., 2014) and the higher level (PEEP = 8 cmH_2_O) was chosen aiming at counteracting the increased P_ab_, since E_rs_ starts to increase at PEEP levels close to 8 cmH_2_O (**Figure 1A**) in the CTRL group. It could be suggested that at this level overdistension might occur, as previously reported (Formenti et al., 2012).

In CTRL group, we obtained the expected E_rs_ vs. PEEP titration curve profile (**Figure 2A**), with a flat region of PEEP of minimal E_rs_, which corresponds to the best balance between tidal recruitment and hyperinflation, higher levels of PEEP leading to overdistention and lower ones to excessive derecruitment (Carvalho et al., 2006, 2008). During the ventilation protocol, as expected, E_rs_ was lower with PEEP = 3 cmH_2_O than with PEEP = 8 cmH_2_O (**Figure 3**), probably because there is no chest wall restriction associated with some degree of lung overdistension (Regli et al., 2012; Runck et al., 2012). Possibly the upper low compliance part of the PV curve was reached.

In PPT group, we obtained almost constant E_rs_ as a function of PEEP (**Figure 2B**). During the ventilation protocol, E_rs_ was always higher than in CTRL rats, suggesting that lung volume was reduced due to abdominal hypertension (**Figures 3**, **4B**), likely leading to alveolar derecruitment. Probably, the balance between P_ab_ and PEEP was not sufficient to recruit the lung (Regli et al., 2012; Runck et al., 2012; Cortes-Puentes et al., 2013). Additionally, **Figure 1B** shows that elastance was constant throughout the total PV curve.

In PPT-RC group, E_rs_ was always higher than in the other groups independently of PEEP level. Possibly, the increased P_ab_ associated with chest wall restriction promoted an important fall in lung volume (**Figure 4C**) that augmented E_rs_ even further. PEEP titration showed a decreasing E_rs_ with increasing pressures (**Figure 2C**), and PEEPmin-_Ers_ corresponded to the highest PEEP step. This suggests that the range of PEEP tested was too narrow, and that the real PEEPmin-_Ers_ is likely to be higher (Loring et al., 2010). E_rs_ was lower with PEEP = 8 cmH_2_O than with PEEP = 3 cmH_2_O during the ventilation protocol (**Figure 3**), likely suggesting some degree of recruitment, as depicted in **Figure 1C**. This is in line with a study on patients with acute respiratory distress syndrome that found a link between the E_w_ increase caused by prone position and the improvement in oxygenation, possibly caused by lung recruitment (Pelosi et al., 1998).

### CT Scan Images

A decrease in TLV was observed by CT scan in PPT group and even further in PPT-RC rats compared to CTRL animals (**Figure 4**). Increasing PEEP from 3 to 8 cmH_2_O led to a significant increase in TLV only in CTRL group. In CTRL group, a high amount of lung units were normally aerated at PEEP = 3 cmH_2_O and a larger PEEP increased aeration. During tidal breathing, CT disclosed a leftward shift of the histogram, suggesting ongoing hyperinflation (**Figure 5A**). This finding reinforces a previously reported association between E_rs_ and imaging evidences of hyperinflation (Carvalho et al., 2006, 2008; Suarez-Sipmann et al., 2007).

In PPT and PPT-RC groups, increasing in chest wall elastance resulted in an overall loss of aeration. In PPT group, increasing PEEP led to a higher contribution of the fraction volume of the apex to TLV (**Figure 6D**), in agreement with the hypothesis that a normal rib cage elastance could allow lung inflation in the regions far from the diaphragm (Regli et al., 2012). Accordingly, with the rib cage restricted (PPT-RC group), an increase in PEEP from 3 to 8 cmH_2_O augmented the contribution of air volume relative to TLV in the middle zones (ventro-dorsal and cephalo-caudal). Using this level of PEEP in animals with normal volume status and cardiac function, we think that this result should not be attributed to a change in intrathoracic and pulmonary blood volume (Luecke et al., 2004; Luecke and Pelosi, 2005). Moreover, the middle ventro-dorsal zone increased its tissue and gas contribution to TLV and the middle cephalocaudal zone tended to follow the same pattern (*p* = 0.053), suggesting lung recruitment closer to the diaphragm. As aforementioned, a PEEP level above 8 cmH_2_O could have further recruited the lung and improved TLV (Loring et al., 2010).

### Limitations

Our study has some limitations. Firstly, the direct clinical application of our findings should be carefully considered owing to differences between rat and human respiratory systems, such as the higher heterogeneity of lung expansion of the latter. Secondly, our protocol design did not include a higher PEEP, which could have led to larger lung recruitment in PPT-RC group. Thirdly, technical issues (esophageal catheter displacement and repositioning due to PEEP) prevented us to measure esophageal pressure and we were unable to evaluate the independent contributions of E_L_ and E_w_ to E_rs_. Fourthly, in a clinical perspective, without an estimation of pleural pressure or an assessment of cardiac function and hemodynamics, we cannot conclude about heart–lung interactions. In the same line, we did not evaluate oxygenation, which could be affected by ventilation/perfusion mismatch resulting from changes in distribution of both ventilation (regional lung mechanics) and perfusion (possible redistribution of blood volume between body compartments and within the lungs). As a last note, our normal-lung rats do not represent patients with respiratory disease, which can further increase lung mechanics heterogeneity.

### Clinical Applications

Observing the mechanical aspects of the interaction of pneumoperitoneum, ribcage elastance and PEEP, the present study confirms our hypothesis that during pneumoperitoneum PEEP alveolar recruitment potential depends on rib cage elastance. We could speculate that, excluding clinical contraindications, patients with a stiffer or heavy ribcage would benefit from the application of high levels of PEEP, while individuals with a normal chest wall compliance would not, or, worse, their lungs could become even hyperinflated. Consequently, the same PEEP value cannot be used in all subjects.

## Conclusion

Rib cage elastance might determine PEEP recruitment potential in the face of abdominal hypertension.

## Author Contributions

LC, MdA, AC, and WZ substantial contributions to the conception or design of the work. LC, MdA, GM-R, RO, TB, NC, AC, and WZ substantial contributions to the acquisition, analysis, or interpretation of data for the work. LC, MdA, GM-R, NC, UL, AC, and WZ drafting the work or revising it critically for important intellectual content. LC, MdA, GM-R, RO, TB, NC, UL, AC, and WZ final approval of the version to be published. LC, MdA, GM-R, RO, TB, NC, UL, AC, and WZ agreement to be accountable for all aspects of the work in ensuring that questions related to the accuracy or integrity of any part of the work are appropriately investigated and resolved.

## Conflict of Interest Statement

The authors declare that the research was conducted in the absence of any commercial or financial relationships that could be construed as a potential conflict of interest.

## Abbreviations

CT scan: computed tomography scan
E_L_: lung elastance
E_rs_: respiratory system elastance
E_w_: chest wall elastance
FiO_2_: inspired oxygen fraction
I:E: inspiratory to expiratory time ratio
PaO_2_/FiO_2_: arterial oxygen partial pressure to inspired oxygen fraction ratio
P_ab_: intra-abdominal pressure
P_aw_: airway pressure
PEEP: positive end-expiratory pressure
PEEPmin-_Ers_: PEEP at minimal elastance
PV curve: pressure-volume curve
ROI: region of interest
RR: respiratory rate
TLC: total lung capacity
TLV: total lung volume
V_T_: tidal volume
